# Characterisation and expression of microRNAs in developing wings of the neotropical butterfly *Heliconius melpomene*

**DOI:** 10.1186/1471-2164-12-62

**Published:** 2011-01-26

**Authors:** Alison K Surridge, Sara Lopez-Gomollon, Simon Moxon, Luana S Maroja, Tina Rathjen, Nicola J Nadeau, Tamas Dalmay, Chris D Jiggins

**Affiliations:** 1 Department of Zoology, University of Cambridge, Downing Street, Cambridge. CB2 3EJ. UK; 2 School of Biological Sciences, University of East Anglia, Norwich. NR4 7TJ. UK; 3 School of Computing Sciences, University of East Anglia, Norwich. NR4 7TJ. UK; 4 Williams College, Department of Biology, Williamstown MA 01267. USA

## Abstract

**Background:**

*Heliconius *butterflies are an excellent system for studies of adaptive convergent and divergent phenotypic traits. Wing colour patterns are used as signals to both predators and potential mates and are inherited in a Mendelian manner. The underlying genetic mechanisms of pattern formation have been studied for many years and shed light on broad issues, such as the repeatability of evolution. In *Heliconius melpomene*, the yellow hindwing bar is controlled by the *HmYb *locus. MicroRNAs (miRNAs) are important post-transcriptional regulators of gene expression that have key roles in many biological processes, including development. miRNAs could act as regulators of genes involved in wing development, patterning and pigmentation. For this reason we characterised miRNAs in developing butterfly wings and examined differences in their expression between colour pattern races.

**Results:**

We sequenced small RNA libraries from two colour pattern races and detected 142 *Heliconius *miRNAs with homology to others found in miRBase. Several highly abundant miRNAs were differentially represented in the libraries between colour pattern races. These candidates were tested further using Northern blots, showing that differences in expression were primarily due to developmental stage rather than colour pattern. Assembly of sequenced reads to the *HmYb *region identified hme-miR-193 and hme-miR-2788; located 2380 bp apart in an intergenic region. These two miRNAs are expressed in wings and show an upregulation between 24 and 72 hours post-pupation, indicating a potential role in butterfly wing development. A search for miRNAs in all available *H. melpomene *BAC sequences (~ 2.5 Mb) did not reveal any other miRNAs and no novel miRNAs were predicted.

**Conclusions:**

Here we describe the first butterfly miRNAs and characterise their expression in developing wings. Some show differences in expression across developing pupal stages and may have important functions in butterfly wing development. Two miRNAs were located in the *HmYb *region and were expressed in developing pupal wings. Future work will examine the expression of these miRNAs in different colour pattern races and identify miRNA targets among wing patterning genes.

## Background

Neotropical butterflies of the genus *Heliconius *provide striking examples of both divergence and convergence in their wing colour patterns. Distributed throughout the tropical forests of central and southern America, they signal their distastefulness to predators through brightly coloured wings. Many species take part in Müllerian mimicry 'rings', where multiple species converge in wing pattern and thereby benefit through protection from predators [1]. Wing patterns are also used in courtship and mate recognition, meaning they are both adaptive and contribute to genetic isolation and speciation [2,3]. Genetic crosses have shown that most phenotypic wing pattern and colour variation is controlled by a few Mendelian loci [4,5]. For example, in *H. melpomene*, genes in linkage group 15 control the yellow and white pattern elements (*HmYb/Sb/N*) and genes in linkage group 18 control red pattern elements (*HmB/D*) [6-8]. The *HmYb *locus, which controls the presence or absence of a hindwing yellow bar, is orthologous to *Cr *in the mimetic species *H. erato*. The loci are found in the same genomic location in these two species and interestingly, also in that of the *P *supergene locus of *H. numata *(which controls a polymorphic whole-wing patterning system) [9]. This suggests that in *Heliconius *the same genetic loci are involved in the repeated evolution of adaptive traits.

In other butterfly species, such as *Bicyclus anynana*, conserved developmental pathways appear to have been co-opted to a role in development of wing pattern elements like eyespots [10,11]. Key transcription factors are involved, such as Notch, Hedgehog and Engrailed [12-14], which have possibly evolved their new role through *cis*-regulatory changes. The developmental basis of wing colour patterning in *Heliconius *has yet to be elucidated. Positional cloning and sequencing of the *HmYb *and *HmB/D *loci and their orthologous loci in *H. erato *have revealed genes that have not been implicated previously in butterfly wing patterning [15-17]. Work is ongoing to further identify the switch genes within the *HmYb *and *HmB/D *regions using population genetics and gene expression approaches. Genetic changes at these switch genes among different colour pattern races are likely to involve *cis*-regulatory or coding sequence changes, changes to post-transcriptional control or a combination of these. MicroRNAs (miRNAs) are important post-transcriptional regulators of gene expression that have been particularly implicated in the fine-tuning of transient and complicated developmental processes. Therefore, they could have a role in the regulation and development of wing patterning that occurs during larval/pupal transitional stages in butterflies.

miRNAs are 19-25 nucleotides long, endogenously expressed non-coding RNAs (for a review see [18]). In animals, mature miRNAs are derived from transcribed hairpin structures (pre-miRNAs) of around 70-90 nucleotides in length that are processed by specialised proteins (Dicer proteins). One strand of the resulting miRNA duplex is incorporated into the RNA-induced silencing complex (RISC). Post-transcriptional silencing is then mediated by binding of this complex to the 3' UTR of the target messengerRNA (mRNA), which causes degradation or translational repression of the gene. Because the 5' seed sequence (at nucleotide positions 2-8) that determines the miRNA and mRNA pairing is just seven nucleotides long in animals [19], one miRNA can have many potential targets and each mRNA can be targeted by more than one miRNA [20].

Some miRNAs are remarkably conserved across distant orders, suggesting conserved evolutionary functions. For example, over half of *Caenorhabditis elegans *miRNAs share sequence homology with those found in human and *Drosophila *[21]. One of the first miRNAs to be discovered, *let-7*, appears to act as an evolutionary conserved developmental timer, with temporal expression patterns being co-ordinated with progression to an adult fate [22]. Loss of *let-7 *function in *Drosophila *leads to widespread defects during metamorphosis, including small wings [23]. Several other studies have shown a key role for miRNAs in metamorphosis and many miRNAs differ in their expression patterns across life stages [24-27]. In the hemimetabolan insect *Blattella germanica*, prevention of miRNA processing by silencing of *Dicer-1 *inhibits metamorphosis, with individuals retaining nymphoid features [28]. Newly emerged miRNA genes have been detected in *Drosophila *and these genes seem to be evolving adaptively and sometimes rapidly [29,30]. Hence, in addition to performing conserved roles, miRNAs could also be involved in the fine-tuning of gene expression patterns underlying the evolution of novel phenotypic traits.

In this study we generated and sequenced small RNA libraries from mixed larval and pupal wings of two colour pattern races of *H. melpomene*; *H. m. rosina*, which has the yellow hindwing bar encoded by the *HmYb *locus and *H. m. melpomene*, which does not. Our aims were to characterise the first miRNAs in *Heliconius *butterflies, to examine differences in expression between two colour pattern races and to identify miRNAs encoded within the *HmYb *region and elsewhere in the genome.

## Results

### *miRNA characterisation in *Heliconius

**Figure 1:**
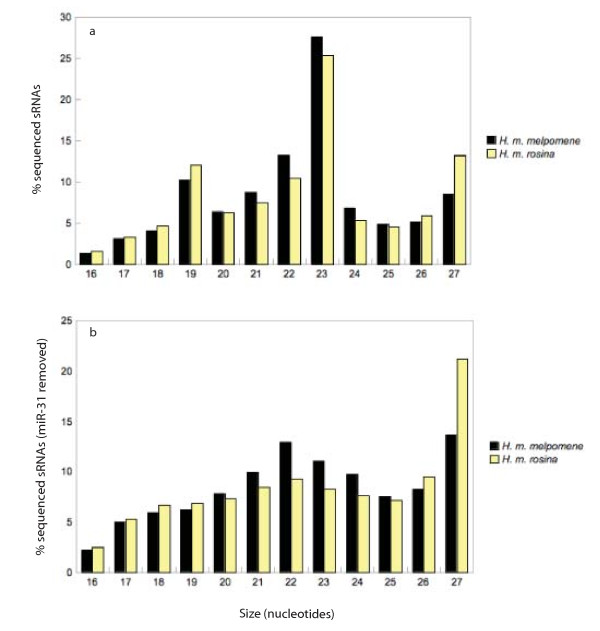
**Size distribution of sequenced small RNAs in *Heliconius melpomene melpomene *(black bars) and *H. m. rosina *(yellow bars)**. % of sequenced reads for each nucleotide size class are given both with (a) and without (b) the most abundant miRNA, miR-31.

A total of 6,895,260 processed sequences were obtained from the small RNA libraries developed for both *Heliconius *races; with more reads being obtained for *H. m. melpomene *(3,967,516) than for *H. m. rosina *(2,927,744). These data have been submitted to the NCBI Gene Expression Omnibus (GEO) [31,32] under accession number GSE23292. Sequences were between 16 and 27 nucleotides in length, with 23 nucleotides being the most abundant length for both races (Figure 1). A comparison of these sequences to miRNAs housed in miRBase (release 15) revealed 142 different sequences with homology to previously identified miRNAs. 49.06% and 45.91% of small RNA sequences were identified as miRNAs for *H. m. melpomene *and *H. m. rosina *respectively. The remaining sequences are likely to include as yet unidentified *Heliconius *miRNAs and other types of small regulatory RNAs. A full list of detected miRNAs, along with normalised abundances for each colour pattern race is given in Additional file 1. The sequences obtained for each race along with read counts are given in Additional file 2 (*H. m. melpomene*) and Additional file 3 (*H. m. rosina).*

#### Expression of miRNAs

**Figure 2:**
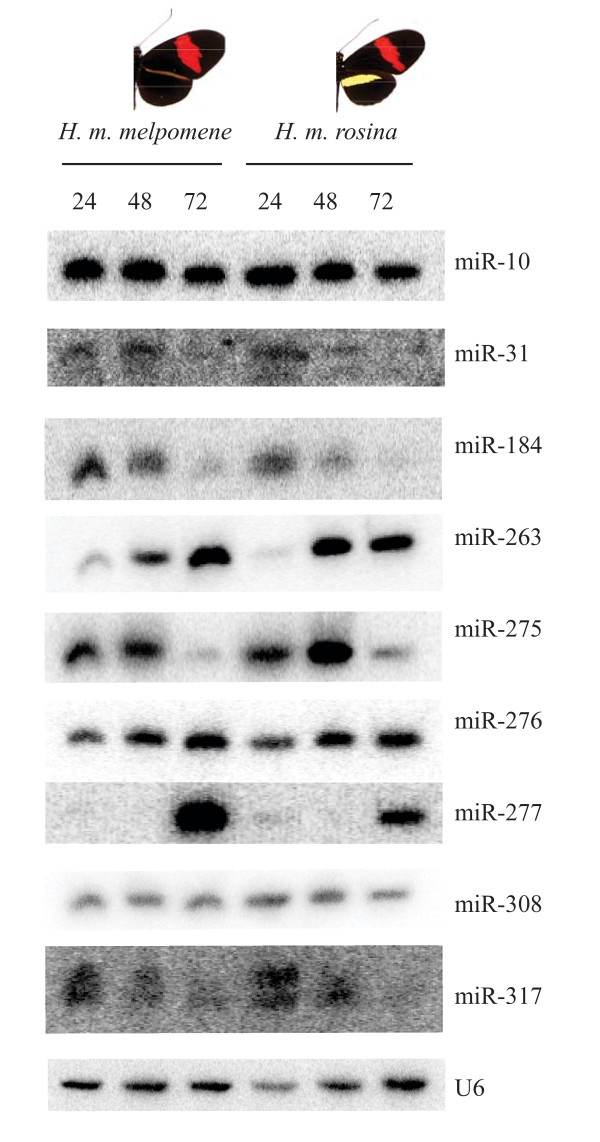
**Northern blots of nine *Heliconius *miRNAs in hindwing tissue from two colour pattern races collected at 24, 48 and 72 hours post-pupation**. Blots were also run for forewing and thorax tissue collected at the same time and all gels were run twice (shown in Additional file 4). Probes are compared to a U6 non-coding small nuclear RNA loading control.

By far the most abundant miRNA, miR-31, accounts for > 50% of all sequenced miRNAs in both colour pattern races. Altogether, 79 miRNAs were present at more than five reads per million sequenced for at least one race and 55 had more than five reads per million for both races. Considering only these 55 miRNAs, 40 had differences in abundance greater than 20% between the two races. Nine of these (chosen for showing large differences in abundance and/or high abundance) were subject to Northern blot analysis across time-staged pupal hindwing development (Figure 2). For each race, hindwing tissue collected from two biological replicates at 24, 48 and 72 hours (± 30 minutes) post-pupation was compared to forewing and thorax tissue collected at the same time (shown in Additional file 4) and to a U6 non-coding small nuclear RNA loading control. In all cases the nine miRNAs were expressed in the three tissue types, i.e. none were either wing, or hindwing specific in their expression. miR-31, miR-10 and miR-308 showed a ubiquitous pattern of expression across stages and races. miR-276 showed an upregulation in both races at 48 and 72 hours compared to 24 and this difference seems to be more pronounced in forewings than hindwings (Additional file 4). For all other miRNAs, expression patterns varied across developmental stage and were mirrored in hindwings and forewings of both races, but not thorax. miR-184 and miR-317 were up-regulated at 24 and 48 hours compared to 72; miR-263 was up-regulated at 48 and 72 hours compared to 24; miR-275 was up-regulated at 48 hours compared to 24 and 72 and miR-277 was up-regulated at 72 hours compared to 24 and 48.

### *Identification of miRNAs in *HmYb

**Figure 3:**
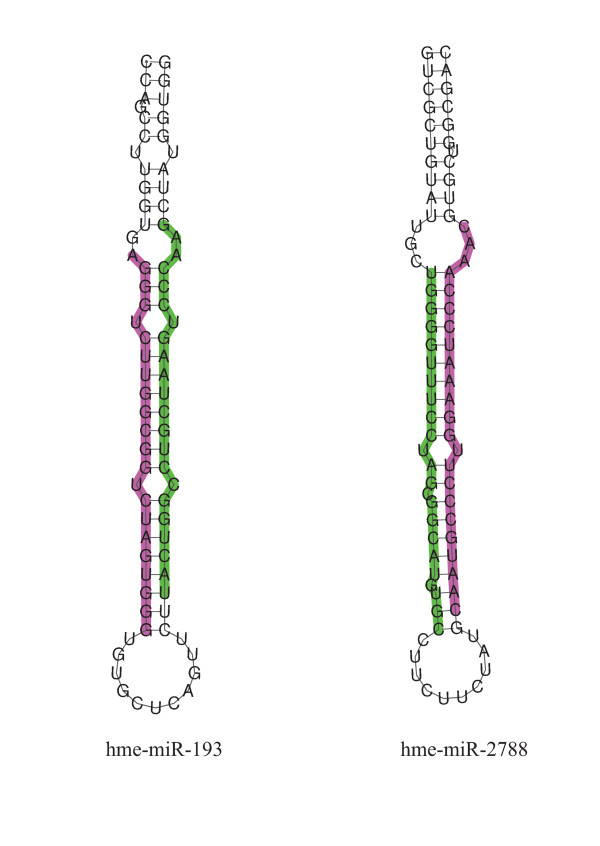
**Predicted stem-loop structures for two miRNAs, hme-miR-193 and hme-miR-2788 found at the *HmYb/Sb *locus**. miRNA sequence is given in green with the star sequence shown in pink.

**Figure 4:**
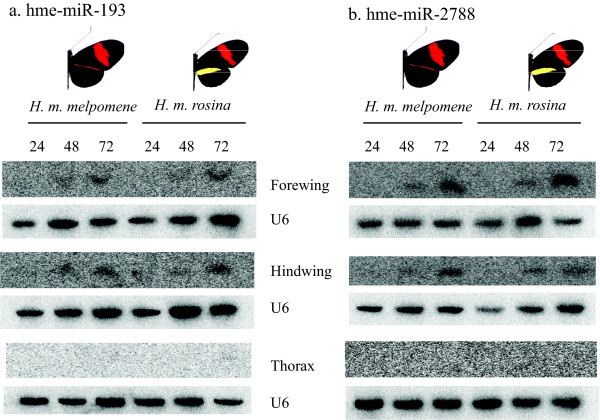
**Northern blots of a. hme-miR-193 and b. hme-miR-2788 in forewing, hindwing and thorax tissue from two colour pattern races collected at 24, 48 and 72 hours post-pupation.** Probes are compared to a U6 non-coding small nuclear RNA loading control.

When short RNA sequence reads were compared to all available *H. melpomene *BAC sequences (currently this consists of ~ 2.5 Mb and includes the *HmYb*/*Sb *and *HmB/D *regions [15,17]) two miRNAs were identified. A comparison of these sequences to miRNAs in miRBase identified them as hme-miR-193 and hme-miR-2788. miR-193 is a conserved miRNA found in mammals, birds, fish and insects. miR-2788 is a newly described miRNA, identified at this time only in *B. mori *[33]. Their predicted stem-loop structures are shown in Figure 3 and an alignment of sequenced reads to the BAC sequence, along with each read count are given in Additional file 5. Interestingly, they are located just 2380 bp apart in the *HmYb *region, in an intergenic location between genes HM00025; a putative member of the *fizzy *family, and HM00026; a putative homologue of *poly(A)-specific ribonuclease *(*PARN*). The butterfly hme-miR-2788 has three mismatches with its closest sequence, found in *B. mori*. Sequence abundances per million reads calculated allowing for these three mismatches between our sequences and those of mature miRNAs present in miRBase (and not two as shown in Additional file 1) were 8.07 and 4.44 for hme-miR-193 and 141.64 and 79.24 for hme-miR-2788, for *H. m. melpomene *and *H. m. rosina *respectively. Therefore both showed a 1.8-fold increase in normalised abundance in *H. m. melpomene *compared to *H. m. rosina*. Northern blot analyses of these two miRNAs show that they are both expressed in forewings and hindwings, with no expression detected in thorax (Figure 4). There was no evidence that they were differentially expressed between colour pattern races, however both show an upregulation from 24 to 72 hours post-pupation. A further search for predicted *Heliconius *miRNAs within the BAC sequences did not identify any new miRNAs, although this analysis did again identify the two miRNAs described above as well as their star sequences (the 'passenger' strand of the pre-miRNA duplex that is not incorporated into the RISC).

## Discussion

### Characterisation of butterfly miRNAs

Here we identify the first miRNAs in butterflies and characterise their expression in developing larval and pupal wings. The size distribution of sequenced reads showed peaks at 23 nucleotides for both *Heliconius *races. Average miRNA length is 22 nucleotides in animals [18]. Using similar deep sequencing techniques, other studies on insects show small RNA sequence size distributions with a peak at 22 nucleotides [34-36] and miRNA sequence sizes appear to peak at 20-22 nucleotides in *B. mori *[24]. In our data the peak at 23 nucleotides is due to miR-31, which is most highly abundant as a 23mer in both races. If this miRNA is excluded, both races show peaks at 22 nucleotides, with another peak at 27 nucleotides that possibly represents Piwi-interacting RNAs; piRNAs (Figure 1).

Sequences were identified as miRNAs only if they shared homology with other miRNAs deposited in miRBase, or if their precursor sequences were identified in *Heliconius *genomic DNA. This approach identified 142 butterfly miRNAs. Recently deep sequencing, or a combination of deep sequencing and bioinformatics has identified 149 miRNAs in the pea aphid, *Acyrthosiphon pisum *[34], and 65 and 77 miRNAs in *Aedes *and *Culex *mosquitoes respectively [35]. Two different studies of *B. mori *yielded 101 conserved and 14 novel miRNAs [24] and 257 miRNAs, of which 202 are described as unique to this species [33]. At this time, 171 *Drosophila melanogaster *miRNAs have been deposited in miRBase. All of our identified miRNAs show homology to other miRNAs in miRBase, as a search of genomic DNA sequences failed to reveal any predicted *Heliconius *specific miRNA precursor sequences. However, only 2.5Mb of genomic BAC clone sequence is available at this time. The completion of the *Heliconius *genome-sequencing project in the near future will facilitate the identification of further miRNAs. These could include butterfly or *Heliconius *specific miRNAs, or miRNAs that were not detected in this study as they are not expressed in wings.

### Expression of butterfly miRNAs in developing wings

The most highly abundant miRNAs, miR-31, miR-184 and miR-263 account for around 60% of detected miRNAs in both races. All three of these miRNAs are also highly conserved and may have important roles in butterfly wing development. Remarkably, miR-31 represented 51% and 56% of all miRNAs sequenced in *H. m. melpomene *and *H. m. rosina *respectively and was expressed in all tissues and developmental stages examined. This miRNA has been best studied in humans, where its dysregulation is implicated in breast, lung, colorectal and head and neck cancers [37-40]. In *Drosophila *it is expressed in a pair rule pattern of 14 stripes, in the anterior endoderm and the hindgut [41]. miR-31 depleted embryos complete development, but have severe segmentation defects including abnormal cuticle patterns and a complete loss of alternating segments [42]. In *B. mori *it shows highest expression during newly hatched larva and moulting larval stages [26] leading to the suggestion that it controls epithelial metabolism during moulting. The very high abundance of this miRNA in *Heliconius *during 5^th ^larval instar and pupal developmental stages suggests a role in butterfly metamorphosis.

In the *Drosophila *embryo, miR-184 is expressed in the central nervous system, brain and imaginal discs [43]. Expression continues into the adult stages and it is also expressed throughout the life cycle of *B. mori *[25] and *Aedes *and *Culex *mosquitoes [44]. It is the most highly expressed miRNA in the mosquito *A. aegypti *[35]. Hence this miRNA may have a general function in insects. However, it shows a decreasing pattern of expression from 24 to 72 hours post-pupation in both races of *Heliconius *(this pattern is not observed for thorax) indicating it may possibly have a more specialised role in butterfly wing development.

miR-263 is preferentially and abundantly expressed in *B. mori *pupae compared to adults [24]. In *Drosophila *it appears to have a conserved affiliation with sensory organ differentiation and is expressed in peripheral sense organs [45]. *Drosophila *deletion mutants show a sporadic loss of sensory bristles, revealing the role of this miRNA in ensuring the developmental 'robustness' of patterning processes (for example by protecting interommatidial bristles during the programmed cell death that refines the pattern of the pupal retina). Intriguingly, this miRNA increases in expression dramatically in *Heliconius *between 24 and 48 hours post-pupation in both forewings and hindwings. Insect sensory bristles and lepidopteron wing scales are widely believed to be homologous structures (with scales being evolutionarily derived from bristles) and share both a specification mechanism and a Notch-mediated lateral inhibition mechanism of patterning [46]. Early scale-forming cell determination occurs 16 hours after pupation, hence the timing of expression of miR-263 in *Heliconius *could be suggestive of a shared role in the developmental pathway of insect sensory organ bristle and lepidopteron wing scale cell patterning.

Most of the miRNAs examined in this study showed dynamic changes in expression pattern throughout pupal wing development, indicating that miRNAs in general have an important role in this process. Changes were mirrored in forewings and hindwings of both colour pattern races (with the exception of miR-276) indicating a role for these miRNAs in general wing development rather than in specific colour patterning. This was in contrast to the sequencing results, where differences in normalised read counts of sequences between races indicated that some miRNAs might be differentially regulated in wings with different colour patterns. Great care was taken to standardise the pool of RNA used for sequencing each race, such that each pool contained the same amount of RNA from each developmental stage represented. However, 5^th ^instar larval wings in particular are difficult to stage with a high degree of accuracy and development is both dynamic and very rapid. Therefore it seems likely that most differences in miRNA abundances observed from the sequence data are due to differences in developmental staging. Future studies of the role of miRNAs in wing colour patterning and pigmentation could investigate miRNAs that are less abundantly expressed, using more sensitive methods for analysing changes in expression (such a quantitative PCR) and also examine specific areas of developing wings (such as that destined to become a yellow bar) to look for regional-specific up- or down-regulation of miRNAs. An understanding of the dynamics of miRNA expression in adult wings would also require further work. It seems likely that different miRNAs would be involved than those expressed during wing development, however once scale cells are developed and pigmented the wing tissue may not have high levels of transcriptional activity (scale cells are not replaced if lost or removed and wings generally fade in colour over time).

#### *miRNAs in *HmYb

Two miRNAs, hme-miR-193 and hme-miR-2788, and their star sequences were discovered in the *HmYb *region. These are located together in an intergenic region 276bp downstream of the stop codon of *PARN *(*HM00026*) and may be co-transcribed. Indeed, their sequences are both represented at a 1.8 fold increase in abundance in *H. m. melpomene*. The other flanking gene (*Fizzy*; *HM00025*) shows differences in splicing that associate with wing colour pattern and is a possible candidate gene for the *HmYb *switch locus. In contrast to the nine other miRNAs examined by Northern blot (described above) these miRNAs did not appear to be expressed in thorax tissue. This implies a wing-specific role. The Northern blots show no evidence for differential expression of the two miRNAs between colour pattern races. Furthermore, both are expressed in forewings and hindwings indicating that their expression is not specific to the yellow hindwing bar pattern encoded by *HmYb*. However, they are strongly up-regulated between 24 and 72 hours in wings, when patterning and pigmentation processes are specified. A role for these two miRNAs in wing colour patterning will be the subject of future research.

## Conclusions

Here we present the first butterfly miRNAs and investigate their role in wing development. Several conserved miRNAs are highly expressed in *Heliconius *pupal wings and one, miR-263, could be involved in wing scale cell patterning in a developmental mechanism that is shared with insect sensory organ bristle patterning. Other miRNAs show dynamic changes in expression in developing pupal wings, indicating an important role for miRNAs in this process. This study did not detect changes in expression of miRNAs associated with wing colour pattern, however two miRNAs were detected in the *HmYb *region that contains colour pattern switch loci. These miRNAs are located close together near a gene that associates with colour pattern and are up-regulated in wings between 24 and 72 hours post-pupation. Future work will investigate possible roles for these and other miRNAs in wing colour patterning.

## Methods

### Sample collection

Wild *H. m. rosina *were collected in Gamboa, Panama (09°07'N/79°42'W) and *H. m. melpomene *were collected in Darien, Panama (09°10'N/78°43'W). Individuals for the study were reared in insectaries at the Smithsonian Tropical Research Institute in Gamboa, Panama. Females were allowed to freely oviposit on *Passiflora menispermifolia*. Eggs were collected daily and larvae were raised in individual pots supplied with fresh young leaves of *P. biflora*. For library development, whole hindwings and forewings were dissected from 5^th ^instar larvae and visually staged for development according to standardised laboratory criteria and those outlined in [47] and [48]. For pupae, wings were collected from two developmental stages, early pupae (where no pigments are visible on wings) and mid-melanin (where ommochrome and melanin pigments are visible). Tissues were stored in RNA*later*^® ^(Ambion). For Northern blot analyses forewing, hindwing and thorax tissue samples were collected separately from a total of nine individuals for each of the two races (three individual replicates for each of three time points post-pupation). Dissections were performed at 24, 48 and 72 hours post-pupation (± 30 minutes) using a Leica Stereozoom 4 microscope under RNase free conditions. All tissues were placed in RNA*later*^® ^(Ambion) and stored at -80°C degrees until extraction.

### RNA extraction, miRNA sequencing and Northern blot

Total RNA (including small RNAs) was extracted using the mirVana™ kit (Ambion) according to the manufacturer's recommendations. RNA was quantified and checked for purity and integrity using the Agilent 2100 Bioanalyzer. For each race, 100 μg of total RNA from 11 individuals was pooled such that each pool contained 50% larval and 50% pupal RNA in equal amounts for each developmental stage (the detailed composition for each pool was 4.1% larval stage < 1; 2% larval stage 1-1.75; 2.9% larval stage 2-2.5; 22% larval stage 2.75-3; 19% larval stage > 3; 25% early pupae; 25% mid-melanin pupae). Both larval and pupal wing stages were included as the most dynamic changes in wing development begin occurring during the final (5th) instar and continue throughout pupation. Small RNA fractions of between 19-24 nucleotides were then isolated and used for short RNA library generation as described in [49]. The libraries were sequenced using an Illumina Genome Analyzer II at BaseClear (Leiden, The Netherlands). 2.5 μg of total RNA was used for Northern blot analysis as described previously [50]. Briefly, RNA from two biological replicates for each race/developmental stage was separated on a 15% denaturing polyacrylamide gel and blotted to Hybond NX membranes (Amersham). Expression of small RNAs was assessed by hybridisation to a [P^32^]-labelled (Perkin Elmer, UK) nucleic acid oligonucleotide probe and compared to a U6 loading control. Probe sequences are given in Additional file 6.

### Computational analysis

Raw Illumina reads were processed by first converting FASTQ to FASTA format, and then removing any adaptor sequences with exact matches to the first eight bases of the 3' adaptor. Any sequences without adaptor matches were excluded from further analyses. Normalised miRNA expression levels were compared between the two *Heliconius *races using miRProf [51] allowing up to two mismatches to mature miRNA sequences present in miRBase [52]. miRNA predictions were performed by running miRCat [51] with default parameters on *H. melpomene *BAC sequences retrieved from Genbank (accessions FP236845.2, FP236798.3, CU463862.6, FP102339.6, FP102341.4, FP102340.5, CU367882.5, CT955980.4, CU462842.3, CU681835.4, FP245488.3, CU467808.6, CU672275.5, CU462858.4, CU672261.2, CU467807.6, CR974474.4, CU928265.1, CU856075.2, CU856076.2, CU856074.2, CU525306.3, CU468009.4, CT573313.6). The predicted miRNAs miR-193 and miR-2788 (and their corresponding sequences and star sequences) were submitted to miRBase [52] and named hme-miR-193 and hme-miR-2788 respectively.

## Authors' contributions

AKS conceived of the study, contributed to its design, co-ordination, data analyses and prepared the manuscript. SL-G performed the Northern blots. SM performed the computational analyses. LSM raised pupae and performed time-staged dissections to provide tissue for Northern blots. TR prepared sRNA for Illumina sequencing. NJN raised larvae and pupae for sRNA sequencing and extracted RNA. TD and CDJ conceived of the study, contributed to its design and helped to prepare the manuscript. All authors read and approved the final manuscript.

## Supplementary Material

Additional file 1 ***Heliconius *miRNAs identified by deep sequencing of two colour pattern races**. miRNAs identified in *H. m. melpomene *and *H. m. rosina*. Two or more miRNAs are listed when a sequence matches to two different, but related miRNAs in miRBase (the sequences and read counts of all miRNA variants detected are given in Additional files 2 and 3). Normalised abundance (number of reads per million; calculated allowing for two mismatches to mature miRNA sequences given in miRBase) is given for each race along with fold change (*melpomene*/*rosina*). Click here for file

Additional file 2 **miRNA sequences and read counts detected in *H. m. melpomene***. FASTA file of all identified miRNA sequence variants detected in *H. m. melpomene*. x refers to the number of reads obtained for each sequence. Click here for file

Additional file 3 **miRNA sequences and read counts detected in *H. m. rosina***. FASTA file of all identified miRNA sequence variants detected in *H. m. rosina*. x refers to the number of reads obtained for each sequence. Click here for file

Additional file 4 **Expression of miRNAs in pupal tissue**. Northern blots for miRNAs in *Heliconius *pupal tissue collected at 24, 48 and 72 hours post-pupation (± 30 mins). *H.m.m *= *H. melpomene melpomene*, *H.m.r *= *H. m. rosina*. FW = forewing, HW = hindwing, T = thorax, U6 = U6 loading control. Gels were run for two biological replicates (A and B) for each race and stage collected. Click here for file

Additional file 5 **Identification of hme-miR-193 and hme-miR-2788 in *Heliconius***. Alignments of different sequences of hme-miR-193 and hme-miR-2788, their star sequences and sequence read counts to *Heliconius melpomene *BAC sequence. Click here for file

Additional file 6 **Probe sequences used for Northern blots**. Probe sequences for ten miRNAs analysed by Northern blot. Click here for file

## References

[B1] Langham GM "Specialized Avian Predators Repeatedly Attack Novel Color Morphs of Heliconius Butterflies," Evolution 2004 58 12 2783 2787 15696755 10.1111/j.0014-3820.2004.tb01629.x

[B2] Jiggins CD Naisbit RE Coe RL Mallet J "Reproductive isolation caused by colour pattern mimicry," Nature 2001 411 6835 302 305 10.1038/35077075 11357131

[B3] Jiggins CD Estrada C Rodrigues A "Mimicry and the evolution of premating isolation in Heliconius melpomene Linnaeus," Journal of Evolutionary Biology 2004 17 3 680 691 10.1111/j.1420-9101.2004.00675.x 15149410

[B4] Sheppard PM Turner JRG Brown KS Benson WW Singer MC "Genetics and the Evolution of Muellerian Mimicry in Heliconius Butterflies," Philosophical Transactions of the Royal Society of London. Series B, Biological Sciences 1985 308 1137 433 610 10.1098/rstb.1985.0066

[B5] Mallet J "The Genetics of Warning Colour in Peruvian Hybrid Zones of Heliconius erato and H. melpomene," Proceedings of the Royal Society of London. B. Biological Sciences 1989 236 1283 163 185 10.1098/rspb.1989.0019

[B6] Gilbert LE Eds Boggs CL, Watt WB, Ehrlich PR "Adaptive novelty through introgression in Heliconius wing patterns: evidence for shared genetic "tool box" from synthetic hybrid zones and a theory of diversification," in Butterflies: ecology and evolution taking flight 2003 University of Chicago Press 281 318

[B7] Naisbit RE Jiggins CD Mallet J "Mimicry: developmental genes that contribute to speciation," Evolution & Development 2003 5 3 269 280 10.1046/j.1525-142x.2003.03034.x 12752766

[B8] Kronforst MR Kapan DD Gilbert LE "Parallel Genetic Architecture of Parallel Adaptive Radiations in Mimetic Heliconius Butterflies," Genetics 2006 174 1 535 539 10.1534/genetics.106.059527 16783007 PMC1569775

[B9] Joron M Papa R Beltrán M Chamberlain N Mavárez J Baxter S Abanto M Bermingham E Humphray SJ Rogers J Beasley H Barlow K ffrench-Constant RH Mallet J McMillan WO Jiggins CD "A Conserved Supergene Locus Controls Colour Pattern Diversity in Heliconius Butterflies," PLoS Biology 2006 4 10 e303 10.1371/journal.pbio.0040303 17002517 PMC1570757

[B10] Monteiro A Glaser G Stockslager S Glansdorp N Ramos D "Comparative insights into questions of lepidopteran wing pattern homology," BMC Developmental Biology 2006 6 52 10.1186/1471-213X-6-52 17090321 PMC1654149

[B11] Carroll S Gates J Keys DN Paddock SW Panganiban GE Selegue JE Williams JA "Pattern formation and eyespot determination in butterfly wings," Science 1994 265 5168 109 114 10.1126/science.7912449 7912449

[B12] Keys DN Lewis DL Selegue JE Pearson BJ Goodrich LV Johnson RL Gates J Scott MP Carroll SB "Recruitment of a hedgehog Regulatory Circuit in Butterfly Eyespot Evolution," Science 1999 283 5401 532 534 10.1126/science.283.5401.532 9915699

[B13] Brunetti CR Selegue JE Monteiro A French V Brakefield PM Carroll SB "The generation and diversification of butterfly eyespot color patterns," Current Biology: CB 2001 11 20 1578 1585 10.1016/S0960-9822(01)00502-4 11676917

[B14] Reed RD Gilbert LE "Wing venation and Distal-less expression in Heliconius butterfly wing pattern development," Development Genes and Evolution 2004 214 12 628 634 10.1007/s00427-004-0439-8 15449055

[B15] Ferguson L Lee SF Chamberlain N Nadeau N Joron M Baxter S Wilkinson P Papanicolaou A Kumar S Kee T-J Clark R Davidson C Glithero R Beasley H Vogel H Ffrench-Constant R Jiggins C "Characterization of a hotspot for mimicry: assembly of a butterfly wing transcriptome to genomic sequence at the HmYb/Sb locus," Molecular Ecology 2010 19 1 240 254 10.1111/j.1365-294X.2009.04475.x 20331783

[B16] Counterman BA Araujo-Perez F Hines HM Baxter SW Morrison CM Lindstrom DP Papa R Ferguson L Joron M ffrench-Constant RH Smith CP Nielsen DM Chen R Jiggins CD Reed RD Halder G Mallet J McMillan WO "Genomic Hotspots for Adaptation: The Population Genetics of Müllerian Mimicry in Heliconius erato," PLoS Genet 2010 6 2 e1000796 10.1371/journal.pgen.1000796 20140239 PMC2816678

[B17] Baxter SW Nadeau NJ Maroja LS Wilkinson P Counterman BA Dawson A Beltran M Perez-Espona S Chamberlain N Ferguson L Clark R Davidson C Glithero R Mallet J McMillan WO Kronforst M Joron M ffrench-Constant RH Jiggins CD "Genomic Hotspots for Adaptation: The Population Genetics of Müllerian Mimicry in the Heliconius melpomene Clade," PLoS Genet 2010 6 2 e1000794 10.1371/journal.pgen.1000794 20140188 PMC2816687

[B18] Bartel DP "MicroRNAs: genomics, biogenesis, mechanism, and function," Cell 2004 116 2 281 297 10.1016/S0092-8674(04)00045-5 14744438

[B19] Lim LP Glasner ME Yekta S Burge CB Bartel DP "Vertebrate microRNA genes," Science (New York, N.Y.) 2003 299 5612 1540 10.1126/science.1080372 12624257

[B20] Brennecke J Stark A Cohen SM "Not miR-ly muscular: microRNAs and muscle development," Genes & Development 2005 19 19 2261 2264 10.1101/gad.1363905 16204177

[B21] Ibáñez-Ventoso C Vora M Driscoll M "Sequence Relationships among C. elegans, D. melanogaster and Human microRNAs Highlight the Extensive Conservation of microRNAs in Biology," PLoS ONE 2008 3 7 e2818 18665242 10.1371/journal.pone.0002818 PMC2486268

[B22] Pasquinelli AE Reinhart BJ Slack F Martindale MQ Kuroda MI Maller B Hayward DC Ball EE Degnan B Müller P Spring J Srinivasan A Fishman M Finnerty J Corbo J Levine M Leahy P Davidson E Ruvkun G "Conservation of the sequence and temporal expression of let-7 heterochronic regulatory RNA," Nature 2000 408 6808 86 89 10.1038/35040556 11081512

[B23] Caygill EE Johnston LA "Temporal regulation of metamorphic processes in Drosophila by the let-7 and miR-125 heterochronic microRNAs," Current Biology: CB 2008 18 13 943 950 10.1016/j.cub.2008.06.020 18571409 PMC2736146

[B24] Jagadeeswaran G Zheng Y Sumathipala N Jiang H Arrese EL Soulages JL Zhang W Sunkar R "Deep sequencing of small RNA libraries reveals dynamic regulation of conserved and novel microRNAs and microRNA-stars during silkworm development," BMC Genomics 2010 11 52 10.1186/1471-2164-11-52 20089182 PMC2824724

[B25] Liu S Zhang L Li Q Zhao P Duan J Cheng D Xiang Z Xia Q "MicroRNA expression profiling during the life cycle of the silkworm (Bombyx mori)," BMC Genomics 2009 10 455 10.1186/1471-2164-10-455 19785751 PMC2761947

[B26] Yu X Zhou Q Li S-C Luo Q Cai Y Lin W-C Chen H Yang Y Hu S Yu J "The Silkworm (Bombyx mori) microRNAs and Their Expressions in Multiple Developmental Stages" PLoS ONE 2008 3 8 e2997 10.1371/journal.pone.0002997 18714353 PMC2500172

[B27] Zhang Y Zhou X Ge X Jiang J Li M Jia S Yang X Y Kan Miao X Zhao G Li F Huang Y "Insect-Specific microRNA Involved in the Development of the Silkworm Bombyx mori," PLoS ONE 2009 4 3 e4677 10.1371/journal.pone.0004677 19262741 PMC2650705

[B28] Gomez-Orte E Belles X "MicroRNA-dependent metamorphosis in hemimetabolan insects," Proceedings of the National Academy of Sciences of the United States of America 2009 106 51 21678 21682 10.1073/pnas.0907391106 19966227 PMC2799836

[B29] Lu J Shen Y Wu Q Kumar S He B Shi S Carthew RW Wang SM Wu C-I "The birth and death of microRNA genes in Drosophila," Nature Genetics 2008 40 3 351 355 10.1038/ng.73 18278047

[B30] Lu J Fu Y Kumar S Shen Y Zeng K Xu A Carthew R Wu C-I "Adaptive evolution of newly emerged micro-RNA genes in Drosophila," Molecular Biology and Evolution 2008 25 5 929 938 10.1093/molbev/msn040 18296702 PMC3707409

[B31] Barrett T Troup DB Wilhite SE Ledoux P Rudnev D Evangelista C Kim IF Soboleva A Tomashevsky M Marshall KA Phillippy KH Sherman PM Muertter RN Edgar R "NCBI GEO: archive for high-throughput functional genomic data," Nucl Acids Res 2009 37 1 D885 890 10.1093/nar/gkn764 18940857 PMC2686538

[B32] Edgar R Domrachev M Lash AE "Gene Expression Omnibus: NCBI gene expression and hybridization array data repository," Nucl Acids Res 2002 30 1 207 210 10.1093/nar/30.1.207 11752295 PMC99122

[B33] Liu S Li D Li Q Zhao P Xiang Z Xia Q "MicroRNAs of Bombyx mori identified by Solexa sequencing," BMC Genomics 2010 11 1 148 10.1186/1471-2164-11-148 20199675 PMC2838851

[B34] Legeai F Rizk G Walsh T Edwards O Gordon K Lavenier D Leterme N Méreau A Nicolas J Tagu D Jaubert-Possamai S "Bioinformatic prediction, deep sequencing of microRNAs and expression analysis during phenotypic plasticity in the pea aphid, Acyrthosiphon pisum," 11 281 281 20444247 10.1186/1471-2164-11-281 PMC2880305

[B35] Skalsky RL Vanlandingham DL Scholle F Higgs S Cullen BR "Identification of microRNAs expressed in two mosquito vectors, Aedes albopictus and Culex quinquefasciatus," BMC Genomics 2010 11 119 20167119 10.1186/1471-2164-11-119 PMC2834634

[B36] Wei Y Chen S Yang P Ma Z Kang L "Characterization and comparative profiling of the small RNA transcriptomes in two phases of locust," Genome Biology 2009 10 1 R6 10.1186/gb-2009-10-1-r6 19146710 PMC2687794

[B37] Valastyan S Reinhardt F Benaich N Calogrias D Szász AM Wang ZC Brock JE Richardson AL Weinber RA "A Pleiotropically Acting MicroRNA, miR-31, Inhibits Breast Cancer Metastasis," Cell 2009 137 6 1032 1046 10.1016/j.cell.2009.03.047 19524507 PMC2766609

[B38] Liu C Tsai M Hung P Kao S Liu T Wu K Chiou S Lin S Chang K "miR-31 Ablates Expression of the HIF Regulatory Factor FIH to Activate the HIF Pathway in Head and Neck Carcinoma," Cancer Research 2010 70 4 1635 1644 10.1158/0008-5472.CAN-09-2291 20145132

[B39] Liu X Sempere LF Ouyang H Memoli VA Andrew AS Luo Y Demidenko E Korc M Shi W Preis M Dragnev KH Li H DiRenzo J Bak M Freemantle SJ Kauppinen S Dmitrovsky E "MicroRNA-31 functions as an oncogenic microRNA in mouse and human lung cancer cells by repressing specific tumor suppressors," Journal of Clinical Investigation 2010 120 4 1298 1309 10.1172/JCI39566 20237410 PMC2846041

[B40] Slaby O Svoboda M Fabian P Smerdova T Knoflickova D Bednarikova M Nenutil R Vyzula R "Altered Expression of miR-21, miR-31, miR-143 and miR-145 Is Related to Clinicopathologic Features of Colorectal Cancer," Oncology 2007 72 5 397 402 10.1159/000113489 18196926

[B41] Aboobaker AA Tomancak P Patel N Rubin GM Lai EC "Drosophila microRNAs exhibit diverse spatial expression patterns during embryonic development," Proceedings of the National Academy of Sciences of the United States of America 2005 102 50 18017 18022 10.1073/pnas.0508823102 16330759 PMC1306796

[B42] Leaman D Chen PY Fak J Yalcin A Pearce M Unnerstall U Marks DS Sander C Tuschl T Gaul U "Antisense-Mediated Depletion Reveals Essential and Specific Functions of MicroRNAs in Drosophila Development," Cell 2005 121 7 1097 1108 10.1016/j.cell.2005.04.016 15989958

[B43] Li P Peng J Hu J Xu Z Xie W Yuan L "Localized expression pattern of miR-184 in Drosophila," Molecular Biology Reports 2010 8 1 355 358 10.1007/s11033-010-0115-1 20339929

[B44] Li S Mead EA Liang S Tu Z "Direct sequencing and expression analysis of a large number of miRNAs in Aedes aegypti and a multi-species survey of novel mosquito miRNAs," BMC Genomics 2009 10 581 10.1186/1471-2164-10-581 19961592 PMC2797818

[B45] Christodoulou F Raible F Tomer R Simakov O Trachana K Klaus S Snyman H Hannon GJ Bork P Arendt D "Ancient animal microRNAs and the evolution of tissue identity," Nature 2010 463 7284 1084 1088 10.1038/nature08744 20118916 PMC2981144

[B46] Reed R "Evidence for Notch-mediated lateral inhibition in organizing butterfly wing scales," Development Genes and Evolution 2004 214 1 43 46 10.1007/s00427-003-0366-0 14618402

[B47] Ferguson LC Jiggins CD "Shared and divergent expression domains on mimetic *Heliconius *wings," *Evolution & Development* 2009 11 5 498 512 19754707 10.1111/j.1525-142X.2009.00358.x

[B48] Reed RD Chen P Nijhout HF "Cryptic variation in butterfly eyespot development: the importance of sample size in gene expression studies," Evolution & Development 2007 9 1 2 9 10.1111/j.1525-142X.2006.00133.x 17227362

[B49] Szittya G Moxon S Santos DM Jing R Fevereiro MP Moulton V Dalmay T "High-throughput sequencing of Medicago truncatula short RNAs identifies eight new miRNA families," BMC Genomics 2008 9 593 19068109 10.1186/1471-2164-9-593 PMC2621214

[B50] Pilcher RLR Moxon S Pakseresht N Moulton V Manning K Seymour G Dalmay T "Identification of novel small RNAs in tomato (Solanum lycopersicum)," Planta 2007 226 3 709 717 10.1007/s00425-007-0518-y 17415587

[B51] Moxon S Schwach F Dalmay T MacLean D Studholme DJ Moulton V "A toolkit for analysing large-scale plant small RNA datasets," Bioinformatics 2008 24 19 2252 2253 10.1093/bioinformatics/btn428 18713789

[B52] Griffiths-Jones S Saini HK van Dongen S Enright AJ "miRBase: tools for microRNA genomics," Nucl Acids Res 2008 36 1 D154 158 17991681 10.1093/nar/gkm952 PMC2238936

