# Supplementary material for: Characterisation and expression of microRNAs in developing wings of the neotropical butterfly Heliconius melpomene
Source: BMC Genomics. 2011 Jan 26;12:62. doi: 10.1186/1471-2164-12-62 (PMC3039609; doi:10.1186/1471-2164-12-62)
Supplement: Additional file 1 — Heliconius miRNAs identified by deep sequencing of two colour pattern races. miRNAs identified in H. m. melpomene and H. m. rosina. Two or more miRNAs are listed when a sequence matches to two different, but related miRNAs in miRBase (the sequences and read counts of all miRNA variants detected are given in Additional files 2 and 3). Normalised abundance (number of reads per million; calculated allowing for two mismatches to mature miRNA sequences given in miRBase) is given for each race along with fold change (melpomene/rosina). [file 1471-2164-12-62-S1.DOC]

|  | **Normalised abundance (reads per million)** | | **Fold change** |
| --- | --- | --- | --- |
| **miRNA** | ***H. m. melpomene*** | ***H. m. rosina*** |  |
| bantam | 148.51 | 165.16 | 0.90 |
| bantam; miR-81 | 0.00 | 0.48 | 0.00 |
| let-7 | 4008.72 | 2763.73 | 1.45 |
| let-7; miR-98 | 6.17 | 1.43 | 4.31 |
| miR-1 | 3622.35 | 4347.03 | 0.83 |
| miR-2 | 198.23 | 192.84 | 1.03 |
| miR-7 | 28.60 | 10.98 | 2.60 |
| miR-8 | 1263.78 | 1108.36 | 1.14 |
| miR-9 | 413.03 | 289.74 | 1.43 |
| miR-10 | 5534.74 | 3715.53 | 1.49 |
| miR-10; miR-100 | 0.00 | 1.43 | 0.00 |
| miR-11 | 375.33 | 390.93 | 0.96 |
| miR-12 | 5.85 | 3.34 | 1.75 |
| miR-13 | 13.32 | 31.50 | 0.42 |
| miR-14 | 50.37 | 49.64 | 1.01 |
| miR-15 | 3.90 | 6.21 | 0.63 |
| miR-18 | 0.00 | 6.21 | 0.00 |
| miR-19 | 8.12 | 0.00 | - |
| miR-21 | 1.62 | 0.00 | - |
| miR-22 | 2.60 | 0.48 | 5.42 |
| miR-24 | 9.75 | 0.95 | 10.26 |
| miR-26 | 5.20 | 0.00 | - |
| miR-27 | 16.25 | 0.00 | - |
| miR-29 | 2.92 | 0.00 | - |
| miR-30 | 117.96 | 60.62 | 1.95 |
| miR-31 | 513000.00 | 561000.00 | 0.91 |
| miR-31; miR-72 | 1243.95 | 723.63 | 1.72 |
| miR-31; miR-72; miR-73 | 0.32 | 0.00 | - |
| miR-33 | 0.00 | 1.91 | 0.00 |
| miR-34 | 10.40 | 5.25 | 1.98 |
| miR-71 | 33.47 | 43.44 | 0.77 |
| miR-72 | 431.22 | 242.01 | 1.78 |
| miR-79 | 24.37 | 10.02 | 2.43 |
| miR-87 | 1.30 | 0.00 | - |
| miR-92 | 217.40 | 179.48 | 1.21 |
| miR-99 | 1.30 | 0.00 | - |
| miR-100 | 10.40 | 17.66 | 0.59 |
| miR-103; miR-107 | 13.32 | 7.64 | 1.74 |
| miR-106; miR-17 | 2.60 | 3.34 | 0.78 |
| miR-125 | 2.60 | 4.30 | 0.60 |
| miR-128 | 5.20 | 0.00 | - |
| miR-130 | 2.27 | 14.32 | 0.16 |
| miR-137 | 0.65 | 0.00 | - |
| miR-140 | 13.65 | 12.89 | 1.06 |
| miR-143 | 2.92 | 8.11 | 0.36 |
| miR-146 | 22.75 | 4.30 | 5.29 |
| miR-148 | 0.65 | 0.00 | - |
| miR-181 | 9.42 | 4.30 | 2.19 |
| miR-182 | 8.45 | 0.95 | 8.89 |
| miR-182; miR-263 | 5.85 | 0.00 | - |
| miR-182; miR-183; miR-263 | 0.32 | 0.00 | - |
| miR-183 | 6.50 | 1.43 | 4.55 |
| miR-183; miR-263 | 57.19 | 43.44 | 1.32 |
| miR-183; miR-228; miR-263 | 0.32 | 0.00 | - |
| miR-184 | 29900.00 | 9816.27 | 3.05 |
| miR-190 | 0.97 | 0.00 | - |
| miR-191 | 1.95 | 0.00 | - |
| miR-193 | 23.07 | 16.71 | 1.38 |
| miR-199 | 7.47 | 3.34 | 2.24 |
| miR-200; miR-8 | 0.97 | 0.48 | 2.02 |
| miR-210 | 36.40 | 5.25 | 6.93 |
| miR-214 | 10.40 | 2.39 | 4.35 |
| miR-221 | 4.55 | 3.34 | 1.36 |
| miR-222 | 0.00 | 10.50 | 0.00 |
| miR-228; miR-263 | 44.19 | 24.34 | 1.82 |
| miR-263 | 54100.00 | 42700.00 | 1.27 |
| miR-265 | 13.00 | 0.00 | - |
| miR-266; miR-31 | 0.00 | 0.48 | 0.00 |
| miR-275 | 127.38 | 44.87 | 2.84 |
| miR-276 | 2375.47 | 805.73 | 2.95 |
| miR-277 | 152.41 | 73.99 | 2.06 |
| miR-278 | 60.44 | 37.23 | 1.62 |
| miR-279 | 1222.18 | 1022.92 | 1.19 |
| miR-279; miR-996 | 0.00 | 0.48 | 0.00 |
| miR-281 | 6.50 | 1.91 | 3.40 |
| miR-282 | 2.92 | 0.00 | - |
| miR-301 | 0.97 | 4.30 | 0.23 |
| miR-305 | 77.67 | 57.28 | 1.36 |
| miR-306 | 6568.45 | 5311.24 | 1.24 |
| miR-307 | 1.62 | 0.00 | - |
| miR-308 | 82.22 | 175.18 | 0.47 |
| miR-317 | 1169.54 | 655.85 | 1.78 |
| miR-352 | 0.00 | 0.48 | 0.00 |
| miR-363 | 5.52 | 18.14 | 0.30 |
| miR-365 | 0.00 | 0.95 | 0.00 |
| miR-429 | 1.30 | 0.00 | - |
| miR-456 | 11.70 | 8.59 | 1.36 |
| miR-489 | 4.55 | 0.00 | - |
| miR-739 | 0.00 | 0.48 | 0.00 |
| miR-745 | 485.49 | 419.57 | 1.16 |
| miR-750 | 3.25 | 0.00 | - |
| miR-927 | 0.00 | 0.95 | 0.00 |
| miR-965 | 56.87 | 39.14 | 1.45 |
| miR-970 | 85.79 | 97.85 | 0.88 |
| miR-988 | 2.27 | 1.91 | 1.19 |
| miR-989 | 167.03 | 97.85 | 1.71 |
| miR-993 | 0.00 | 0.48 | 0.00 |
| miR-998 | 10.72 | 2.86 | 3.75 |
| miR-1207 | 0.65 | 0.00 | - |
| miR-1260 | 3.90 | 0.00 | - |
| miR-1274 | 0.97 | 0.00 | - |
| miR-1308 | 13.00 | 56.32 | 0.23 |
| miR-1456 | 22.10 | 8.59 | 2.57 |
| miR-1576 | 0.00 | 0.48 | 0.00 |
| miR-1630 | 0.00 | 2.39 | 0.00 |
| miR-1631 | 0.32 | 0.00 | - |
| miR-1716 | 0.00 | 0.48 | 0.00 |
| miR-1763 | 7.47 | 64.44 | 0.12 |
| miR-1770 | 0.00 | 0.95 | 0.00 |
| miR-1777 | 0.00 | 0.48 | 0.00 |
| miR-1795 | 2.27 | 0.00 | - |
| miR-1895 | 0.65 | 1.43 | 0.45 |
| miR-1937 | 29.25 | 28.64 | 1.02 |
| miR-1939 | 2.60 | 0.00 | - |
| miR-1959 | 3.90 | 0.48 | 8.13 |
| miR-2131 | 4.55 | 3.34 | 1.36 |
| miR-2138 | 822.80 | 909.31 | 0.90 |
| miR-2140 | 0.00 | 2.39 | 0.00 |
| miR-2145 | 1.30 | 0.00 | - |
| miR-2273 | 0.00 | 0.48 | 0.00 |
| miR-2395 | 0.32 | 0.00 | - |
| miR-2428 | 0.32 | 0.48 | 0.67 |
| miR-2478 | 5.20 | 0.00 | - |
| miR-2738 | 3.57 | 0.48 | 7.44 |
| miR-2755 | 3012.39 | 2672.56 | 1.13 |
| miR-2756 | 37.37 | 85.44 | 0.44 |
| miR-2763 | 1.30 | 0.00 | - |
| miR-2765 | 32.82 | 26.73 | 1.23 |
| miR-2766 | 49.72 | 27.21 | 1.83 |
| miR-2767 | 790.63 | 881.15 | 0.90 |
| miR-2768 | 47.44 | 82.10 | 0.58 |
| miR-2779 | 10.40 | 6.68 | 1.56 |
| miR-2788 | 0.00 | 0.48 | 0.00 |
| miR-2954 | 0.97 | 0.00 | - |
| miR-3008 | 0.00 | 1.43 | 0.00 |
| miR-3162 | 1.30 | 0.00 | - |
| miR-3168 | 0.97 | 0.48 | 2.02 |
| miR-3281 | 9.42 | 3.82 | 2.47 |
| miR-4125 | 0.00 | 1.91 | 0.00 |
| miR-4128 | 0.32 | 1.43 | 0.22 |
| miR-4175 | 0.00 | 0.48 | 0.00 |
| miR-4184 | 0.32 | 0.00 | - |
