# Supplementary material for: Characterisation and expression of microRNAs in developing wings of the neotropical butterfly Heliconius melpomene
Source: BMC Genomics. 2011 Jan 26;12:62. doi: 10.1186/1471-2164-12-62 (PMC3039609; doi:10.1186/1471-2164-12-62)
Supplement: Additional file 4 — Expression of miRNAs in pupal tissue. Northern blots for miRNAs in Heliconius pupal tissue collected at 24, 48 and 72 hours post-pupation (± 30 mins). H.m.m = H. melpomene melpomene, H.m.r = H. m. rosina. FW = forewing, HW = hindwing, T = thorax, U6 = U6 loading control. Gels were run for two biological replicates (A and B) for each race and stage collected. [file 1471-2164-12-62-S4.DOC]

miR-31

244872

244872

244872

244872

A

B

FW

U6

U6

U6

HW

T

*H.m.m*

*H.m.m*

*H.m.r*

*H.m.r*


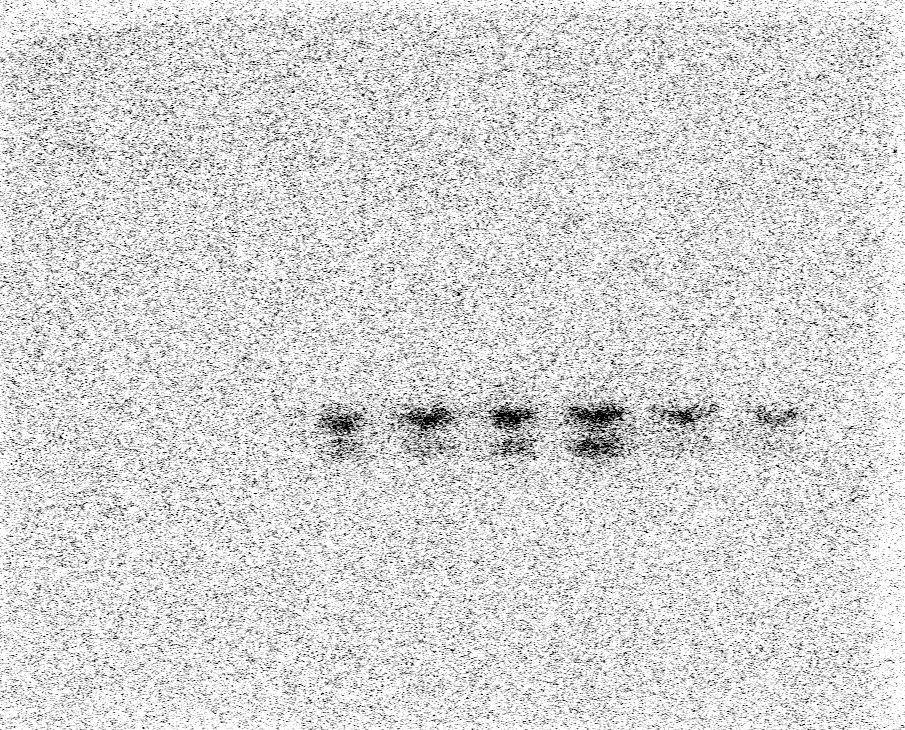

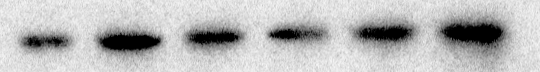

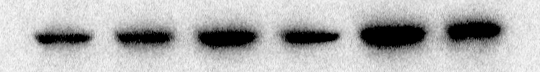

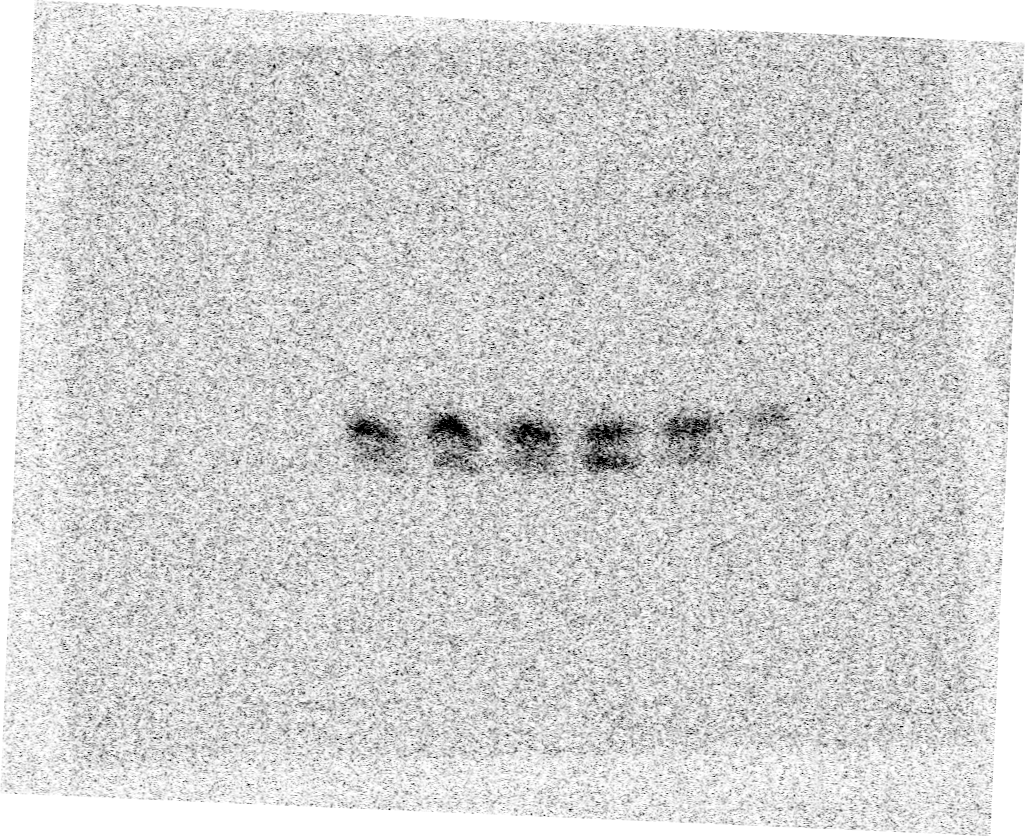

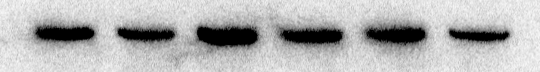

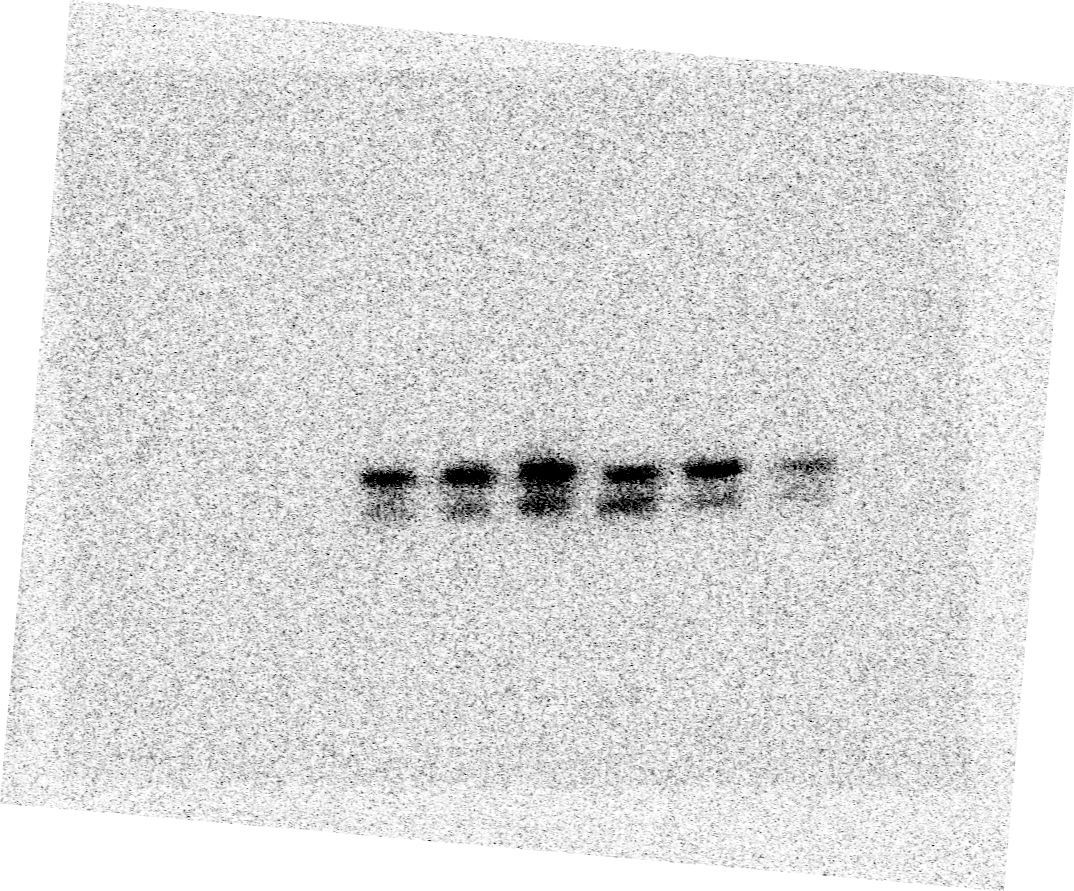

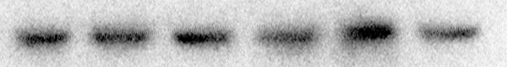

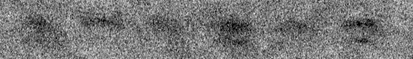

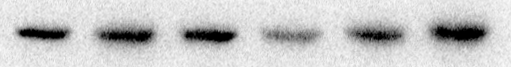

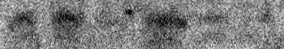

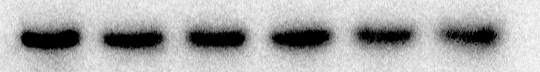

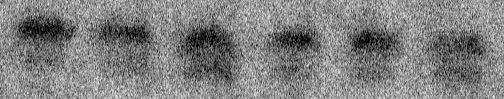


miR-10

244872

244872

244872

244872

A

B

FW

U6

U6

U6

HW

T

*H.m.m*

*H.m.m*

*H.m.r*

*H.m.r*

miR-308

244872

244872

244872

244872

A

B

FW

U6

U6

U6

HW

T

*H.m.m*

*H.m.m*

*H.m.r*

*H.m.r*

miR-276

244872

244872

244872

244872

A

B

FW

U6

U6

U6

HW

T

*H.m.m*

*H.m.m*

*H.m.r*

*H.m.r*

miR-184

244872

244872

244872

244872

A

B

FW

U6

U6

U6

HW

T

*H.m.m*

*H.m.m*

*H.m.r*

*H.m.r*

miR-317

244872

244872

244872

244872

A

B

FW

U6

U6

U6

HW

T

*H.m.m*

*H.m.m*

*H.m.r*

*H.m.r*

miR-263

244872

244872

244872

244872

A

B

FW

U6

U6

U6

HW

T

*H.m.m*

*H.m.m*

*H.m.r*

*H.m.r*

miR-275

244872

244872

244872

244872

A

B

FW

U6

U6

U6

HW

T

*H.m.m*

*H.m.m*

*H.m.r*

*H.m.r*

miR-277

244872

244872

244872

244872

A

B

FW

U6

U6

U6

HW

T

*H.m.m*

*H.m.m*

*H.m.r*

*H.m.r*
