# Supplementary material for: Characterisation and expression of microRNAs in developing wings of the neotropical butterfly Heliconius melpomene
Source: BMC Genomics. 2011 Jan 26;12:62. doi: 10.1186/1471-2164-12-62 (PMC3039609; doi:10.1186/1471-2164-12-62)
Supplement: Additional file 5 — Identification of hme-miR-193 and hme-miR-2788 in Heliconius. Alignments of different sequences of hme-miR-193 and hme-miR-2788, their star sequences and sequence read counts to Heliconius melpomene BAC sequence. [file 1471-2164-12-62-S5.PDF]

hme-miR-2788

```
GTCGCTGTATTGCTGGGGTTTCCTAGCGGCATGTGCCTTCTTCTATGCAATGCCCTTGGAAATCCCAAACGTGCTGGCGAC  Sequence abundance
.............TGGGGTTTCCTAGCGGCAT.................................................  16
.............TGGGGTTTCCTAGCGGCATG................................................  102
.............TGGGGTTTCCTAGCGGCATGT...............................................  234
.............TGGGGTTTCCTAGCGGCATGTG..............................................  306
.............TGGGGTTTCCTAGCGGCATGTGC.............................................  794    hme-miR-2788
.............TGGGGTTTCCTAGCGGCATGTGCC............................................  33
.............TGGGGTTTCCTAGCGGCATGTGCCT...........................................  20
...............................................CAATGCCCTTGGAAATCCCAA.............  2
...............................................CAATGCCCTTGGAAATCCCAAA............  27     hme-miR-2788*
...............................................CAATGCCCTTGGAAATCCCAAAC...........  2
................................................AATGCCCTTGGAAATCCCAAA............  2
```

hme-miR-193

```
CCAGCCTTGGTGAGGGTCTTGGCGGTCTAGTGGGTGTGCTCAGTTCTTACTGGCCTGCTAAGTCCCAAGCTATGGTGG
............AGGGTCTTGGCGGTCTAGT...............................................     1
............AGGGTCTTGGCGGTCTAGTG..............................................     20
............AGGGTCTTGGCGGTCTAGTGG.............................................     30     hme-miR-193*
............AGGGTCTTGGCGGTCTAGTGGG............................................     26
...............................................TACTGGCCTGCTAAGTCCCA...........     2
...............................................TACTGGCCTGCTAAGTCCCAA..........     39
...............................................TACTGGCCTGCTAAGTCCCAAG.........     45     hme-miR-193
```
