# Supplementary material for: Characterisation and expression of microRNAs in developing wings of the neotropical butterfly Heliconius melpomene
Source: BMC Genomics. 2011 Jan 26;12:62. doi: 10.1186/1471-2164-12-62 (PMC3039609; doi:10.1186/1471-2164-12-62)
Supplement: Additional file 6 — Probe sequences used for Northern blots. Probe sequences for ten miRNAs analysed by Northern blot. [file 1471-2164-12-62-S6.DOC]

| **miRNA** | **Probe Sequence (5’ to 3’)** |
| --- | --- |
| miR-10 | ACAAATTCGGATCTACAGGGT |
| miR-31 | TCAGCTATGCCGACATCTTGCCT |
| miR-184 | GCCCTTATCAGTTCTCCGTCCAGT |
| miR-193 | CTTGGGACTTAGCAGGCCAGTA |
| miR-263 | GTGAATTCTTCCAGTGCCATT |
| miR-275 | CGCGCGCTACTTCAGGTACCTGA |
| miR-276 | AGAGCACGGTATGAAGTTCCTA |
| miR-277 | TGTCGTACCAGATAGTGCATTTA |
| miR-308 | CTCGCAGTATTATCCTGTGATT |
| miR-317 | GATACCACCAGCTGTGTTCACT |
| miR-2788 | GCACATGCCGCTAGGAAACCCCA |
| U6 | ATCCTTGCGCAGGGGCCATGCTAA |
